# Presumed non-inflammatory bilateral peripapillary subretinal fibrosis in adolescent patients

**DOI:** 10.1186/s12886-026-05466-5

**Published:** 2026-10-24

**Authors:** Selda Celik Dulger, Mehmet Yasin Teke

**Affiliations:** Department of Ophthalmology, Ministry of Health Ankara Etlik City Hospital, Ankara, Turkey

**Keywords:** Peripapillary subretinal fibrosis, Adolescent retina, Choroidal neovascularization, Optical coherence tomography, Fluorescein angiography

## Abstract

**Background:**

To describe two adolescent patients with bilateral peripapillary subretinal fibrosis (PSF) without overt clinical or angiographic evidence of active intraocular inflammation.

**Case presentation:**

Observational case series of two adolescent patients who underwent comprehensive ophthalmic examination, including color fundus photography, fluorescein angiography (FA), and spectral-domain optical coherence tomography (OCT). Both patients demonstrated bilateral peripapillary subretinal fibrotic lesions with variable macular involvement. In Case 1, the left eye showed macular extension with findings suggestive of a subfoveal choroidal neovascular membrane, associated with intraretinal cystic changes on OCT, while the fellow eye exhibited a peripapillary lesion sparing the macula. In Case 2, the left eye showed macular involvement with marked outer retinal atrophy and increased choroidal hypertransmission, whereas the right eye had an isolated peripapillary fibrotic lesion without foveal involvement. Fluorescein angiography revealed late staining of the fibrotic areas without angiographic signs of active intraocular inflammation; window defect–related hyperfluorescence was observed in areas of atrophy. Visual acuity reduction was observed only in eyes with macular involvement.

**Conclusions:**

These cases describe a presumed non-inflammatory presentation of bilateral peripapillary subretinal fibrosis in adolescent patients. However, an underlying inherited retinal disorder or subclinical inflammatory or infectious disease cannot be completely excluded because the relevant historical investigations could not be verified. Further studies, including genetic and electrophysiological evaluation, are required to better define the underlying mechanisms.

## Introduction

Peripapillary subretinal fibrosis (PSF) is an uncommon retinal finding that has most frequently been described in association with inflammatory chorioretinal disorders, particularly multifocal choroiditis (MFC) and panuveitis [1, 2]. In these conditions, fibrosis often develops at sites of prior inflammatory lesions and may extend toward the peripapillary region or macula, leading to permanent structural damage and irreversible visual loss [3–8]. PSF has also been reported in association with inherited retinal disorders, most notably in patients with biallelic *NR2E3* mutations, where fibrosis may occur as part of a broader spectrum of retinal degeneration including outer retinal atrophy, nummular deposits characteristic of enhanced S-cone syndrome, and helicoid subretinal lesions [9, 10].

Peripapillary subretinal fibrosis has most frequently been described in association with inflammatory or inherited retinal disorders. However, bilateral peripapillary subretinal fibrosis without overt clinical evidence of active inflammation may also be encountered. The present study describes two adolescent patients with bilateral peripapillary subretinal fibrosis without overt clinical or angiographic evidence of active intraocular inflammation. We use the term “presumed non-inflammatory phenotype” to reflect the clinical findings while acknowledging that inflammatory and inherited causes cannot be definitively excluded.

## Case presentation

This study was a retrospective observational case series based on clinical records and multimodal imaging findings of two adolescent patients evaluated at a tertiary referral centre. Two patients were followed for approximately four years with serial clinical examinations and multimodal imaging, including colour fundus photography, fluorescein angiography (FA), and spectral-domain optical coherence tomography (SD-OCT).

Medical history and available clinical records were reviewed for evidence of prior ocular inflammation, infection, trauma, or systemic inflammatory or inherited retinal disease. Because these were historical cases, the available records were incomplete, and the results of genetic testing, electrophysiological evaluation, and systemic laboratory investigations, if performed during the original evaluation, could not be verified. Therefore, inherited retinal disease and subclinical inflammatory or infectious disease could not be definitively excluded.

Patient 1 was a 14-year-old male with a best-corrected visual acuity (BCVA) of 6/7.5 in the right eye and counting fingers at 5 m in the left eye. Anterior segment examination was unremarkable, with no evidence of cells or haze. Fundus examination revealed bilateral peripapillary subretinal fibrosis.

**Fig. 1:**
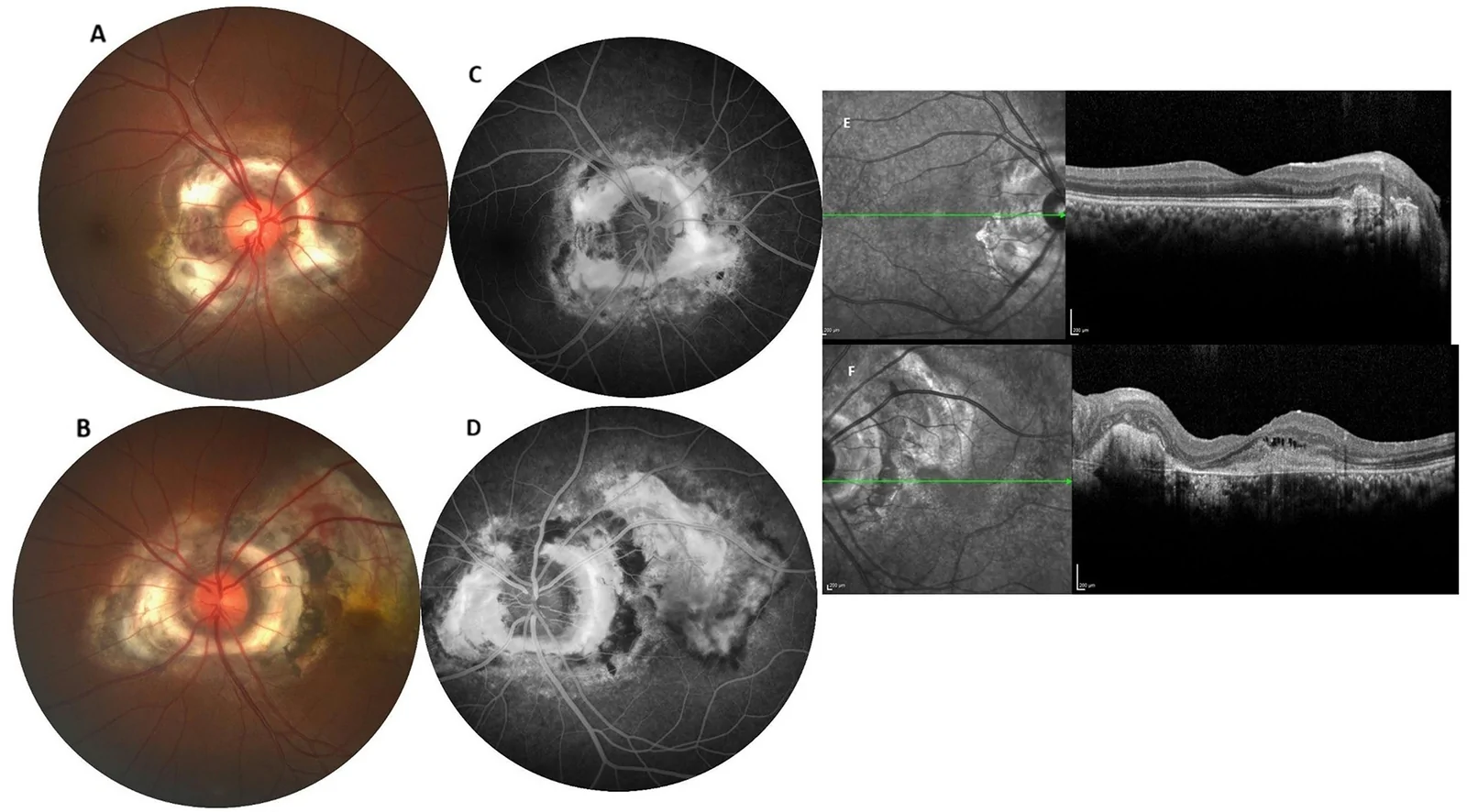
(**A**, **B**) Color fundus photographs showing bilateral peripapillary subretinal fibrotic lesions in Case 1, with macular extension in the left eye and peripapillary-confined involvement in the right eye. (**C**, **D**) Fluorescein angiography of Case 1 demonstrating late staining of the fibrotic areas and blocked fluorescence corresponding to hyperpigmented regions; a hyperfluorescent lesion with features suggestive of choroidal neovascularization is seen in the left eye. (**E**, **F**) Optical coherence tomography (OCT) scans of Case 1 showing a subretinal hyperreflective lesion temporal to the optic disc in the right eye and dome-shaped subretinal hyperreflective lesions with associated intraretinal cystic changes in the left eye

In the left eye, the fibrotic lesion extended from the peripapillary region towards the macula, involving the foveal area, whereas the right eye demonstrated a lesion confined to the peripapillary region. No multifocal chorioretinal scars, punched-out lesions, or signs of active inflammation were observed. Areas of hyperpigmentation were present within the fibrotic region (Fig. 1A, B).

Fluorescein angiography demonstrated late staining of the fibrotic areas, while hyperpigmented regions showed blocked fluorescence. In the macula, a hyperfluorescent lesion surrounded by a hypofluorescent halo was observed, suggestive of a subfoveal choroidal neovascular lesion in the left eye (Fig. 1C, D). Because OCT angiography was not available from the original evaluation, the vascular lesion could not be further classified by OCT-A.

On SD-OCT, the right eye demonstrated a subretinal hyperreflective lesion temporal to the optic disc, with preserved macular architecture. In the left eye, two dome-shaped hyperreflective subretinal lesions were identified, one temporal to the optic disc and the other beneath the fovea. Several small intraretinal cystic spaces were observed beneath the fovea, suggestive of choroidal neovascularisation, while outer retinal tubulation was noted in the peripapillary region (Fig. 1E, F). Two intravitreal anti-vascular endothelial growth factor injections were administered to the left eye. The available historical records did not allow reliable verification of pre- or post-treatment visual acuity, OCT response, injection interval, CNV subtype, or specific recurrence data.

Patient 2 was a 16-year-old female with a BCVA of 6/6 in the right eye and counting fingers at 3 m in the left eye. Slit-lamp examination revealed no signs of anterior segment inflammation. Fundus examination demonstrated bilateral peripapillary subretinal fibrosis.

**Fig. 2:**
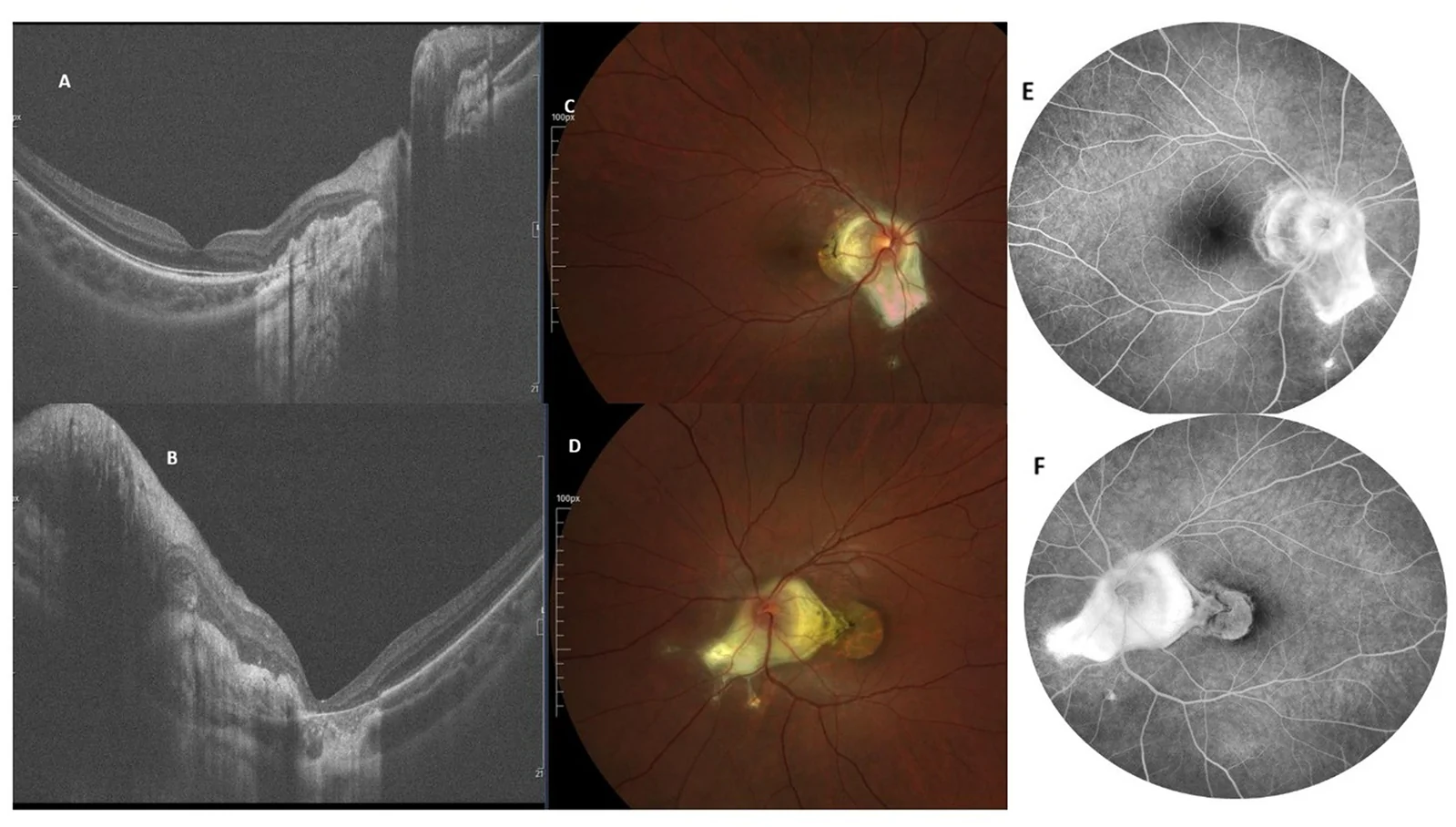
(**A**–**B**) OCT images showing peripapillary subretinal hyperreflective scar tissue and macular outer retinal atrophy with increased choroidal hypertransmission in the left eye; (**C**–**D**) color fundus photographs showing bilateral peripapillary subretinal fibrosis with macular involvement in the left eye; (**E**–**F**) fluorescein angiography images showing late staining of the fibrotic areas and a window defect corresponding to macular atrophy in the left eye

In the left eye, a fibrotic extension originating from the peripapillary region extended towards and involved the macula, where marked macular atrophy was present. The right eye demonstrated a peripapillary fibrotic lesion without macular involvement. No additional chorioretinal lesions or vitreous haze were identified, and no clinically apparent signs of intraocular inflammation were observed (Fig. 2C, D).

Fluorescein angiography revealed late staining of the fibrotic areas, consistent with scar tissue. In the macula of the left eye, hyperfluorescence due to a window defect was observed, without evidence of leakage (Fig. 2E, F).

SD-OCT of the right eye demonstrated subretinal hyperreflective scar tissue located temporal and nasal to the optic disc, without extension into the fovea. The foveal contour and outer retinal layers were preserved.

In the left eye, macular atrophy involving the outer retinal layers beneath the fovea was observed in association with subretinal fibrotic tissue, along with increased choroidal hypertransmission secondary to retinal pigment epithelium atrophy (Fig. 2A, B).

During the approximately four-year follow-up, no clinically apparent enlargement of the lesions, development of new lesions, or progression towards the macula was documented in the available records.

## Discussion

The present cases demonstrate bilateral peripapillary subretinal fibrosis in adolescent patients without overt clinical or angiographic evidence of active ocular inflammation. The consistent peripapillary localisation, bilateral involvement, and absence of accompanying inflammatory signs distinguish these cases clinically from the classical presentation associated with multifocal choroiditis and related inflammatory disorders [1–5]. However, the absence of overt inflammatory findings cannot exclude previous or subclinical inflammation. Accordingly, we consider these cases to represent a possible or presumed non-inflammatory phenotype rather than a definitively established non-inflammatory disease entity.

An important observation in this series is the variability in macular involvement and clinical outcome. Visual impairment was observed exclusively in eyes with macular extension, whereas eyes with lesions confined to the peripapillary region preserved normal visual acuity. This finding indicates that visual prognosis appears to be primarily determined by macular involvement rather than the presence of peripapillary fibrosis itself.

The consistent bilateral localisation and absence of overt inflammatory features across both patients support the possibility that these findings may represent a reproducible clinical phenotype, although a broader spectrum of disease or different underlying mechanisms cannot be excluded.

In Case 1, the presence of subfoveal choroidal neovascularisation raises the question of whether fibrotic changes are causally related to neovascular processes. However, the temporal relationship between fibrosis and choroidal neovascularisation remains uncertain. Given the stable bilateral peripapillary distribution and the absence of inflammatory signs, the suspected choroidal neovascularisation may represent a secondary complication; however, its relationship with the fibrotic changes remains uncertain.

The observed morphological variability between the two patients may reflect different stages of a common underlying process, differences in chronicity, or heterogeneity in pathogenic mechanisms. These cases may represent different manifestations within a broader spectrum of bilateral peripapillary subretinal fibrosis rather than a single established clinical entity.

In inflammatory chorioretinal disorders, subretinal fibrosis is thought to arise from chronic or recurrent damage to the outer retina, retinal pigment epithelium, and choroid, with subsequent activation of fibroblasts, myofibroblasts, and retinal pigment epithelium-derived cells leading to extracellular matrix deposition and scar formation [3–8]. In contrast, the absence of clinical or angiographic evidence of inflammation in the present cases suggests that alternative mechanisms may be involved. These may include primary retinal pigment epithelium dysfunction, localised microvascular insufficiency, or developmental anomalies affecting the peripapillary region.

Peripapillary subretinal fibrosis has also been described in association with inherited retinal disease, particularly in patients with biallelic NR2E3 mutations, where fibrosis may occur alongside outer retinal atrophy and characteristic pigmentary changes [9, 10]. Although no characteristic clinical or multimodal imaging features suggestive of NR2E3-related retinopathy were identified in the present cases, an underlying inherited retinal disorder could not be definitively excluded in the absence of verifiable genetic and electrophysiological testing. Importantly, Buerk et al. [1] described peripapillary subretinal fibrosis as a characteristic finding of multifocal choroiditis and panuveitis, whereas Hassanpoor et al. [9] demonstrated that helicoid peripapillary subretinal fibrosis may also occur as part of an inherited retinal phenotype associated with biallelic NR2E3 mutations. This is particularly relevant in the present adolescent patients, and an underlying inherited retinal disorder cannot be definitively excluded.

The differential diagnosis should therefore include multifocal choroiditis/panuveitis, NR2E3-associated retinal disease, serpiginous choroiditis, and other inherited retinal disorders characterized by outer retinal degeneration, retinal pigment epithelium abnormalities, macular atrophy, or helicoid/peripapillary lesions.

From a clinical perspective, recognising this pattern may help avoid premature classification as inflammatory chorioretinal disease and may reduce unnecessary investigations or inappropriate immunosuppressive treatment. These cases highlight that peripapillary subretinal fibrosis can occur in the absence of overt inflammation and appeared relatively stable during the available follow-up period.

The main limitations of this study include the small sample size and the inability to verify historical genetic, electrophysiological, systemic laboratory, and OCT angiography investigations.

This case series suggests that bilateral peripapillary subretinal fibrosis may represent a possible clinical phenotype in adolescent patients without overt clinical or angiographic evidence of active inflammation. However, inherited retinal disease and subclinical inflammatory or infectious disease cannot be completely excluded. Further studies are required to clarify the pathophysiology and clinical spectrum of this presentation.

## Data Availability

The datasets used or analyzed during the current study are available from the corresponding author upon reasonable request.
